# Anosmia in COVID-19 could be associated with long-term deficits in the consolidation of procedural and verbal declarative memories

**DOI:** 10.3389/fnins.2022.1082811

**Published:** 2022-12-09

**Authors:** Tania Llana, Marta Mendez, Candela Zorzo, Camino Fidalgo, M.-Carmen Juan, Magdalena Mendez-Lopez

**Affiliations:** ^1^Department of Psychology, Faculty of Psychology, University of Oviedo, Oviedo, Spain; ^2^Neuroscience Institute of Principado de Asturias (INEUROPA), Oviedo, Spain; ^3^Instituto de Investigación Sanitaria del Principado de Asturias (ISPA), Oviedo, Spain; ^4^Instituto Universitario de Automática e Informática Industrial, Universitat Politècnica de València, Valencia, Spain; ^5^Department of Psychology and Sociology, University of Zaragoza, Zaragoza, Spain; ^6^IIS Aragón-Instituto de Investigación Sanitaria Aragón, Zaragoza, Spain

**Keywords:** long-COVID, declarative memory, implicit memory, incidental learning, anosmia

## Abstract

**Background and purpose:**

Long-COVID describes the long-term effects of the coronavirus disease 2019 (COVID-19). In long-COVID patients, neuropsychological alterations are frequently reported symptoms. Research points to medial temporal lobe dysfunction and its association with anosmia in long-COVID patients. This study aims to investigate the acquisition and consolidation of declarative and procedural memory in long-COVID patients and to explore whether anosmia is related to these dissociated memory functions.

**Methods:**

Forty-two long-COVID participants and 30 controls (C) were recruited. The sample of long-COVID patients was divided into two groups based on the presence or absence of anosmia, group A and group NA, respectively. Objective performance in verbal declarative memory (Paired-Associate Learning, PAL), procedural memory (Mirror Tracing Test, MTT), general cognitive function (Montreal Cognitive Assessment scale), psychomotor speed, and incidental learning (Digit Symbol Substitution Test) were assessed and compared among the A, NA, and C groups. Long-term retention of PAL and MTT were assessed 24 h after acquisition.

**Results:**

Lower scores in general cognition, psychomotor speed, and sustained attention were found in A and NA compared with C. However, incidental learning, both cue-guided and free-recalled, was diminished in group A compared with C, with no differences with group NA. General cognition and incidental learning were related to declarative memory function exclusively in long-COVID groups. Long-COVID groups presented lower long-term retention of verbal declarative memory than controls in recall tests but no differences in recognition tests. No group differences were found in the acquisition of procedural memory. However, long-term retention of this memory was worse in group A as compared to the NA and C groups, respectively, when errors and time of execution were considered.

**Conclusion:**

Findings support that consolidation of both procedural and declarative memories is more affected than the acquisition of these memories in long-COVID patients, who are also more vulnerable to deficits in delayed recall than in recognition of declarative memories. Deficits in the consolidation of procedural memory and immediate recall of declarative information are especially relevant in long-COVID participants with anosmia. This indicates that anosmia in COVID-19 could be associated with a long-term dysfunction of the limbic system.

## Introduction

Coronavirus disease (COVID-19) is a multisystemic illness caused by the severe acute respiratory syndrome-2 infection (SARS-CoV-2), which can produce various symptoms, including upper respiratory symptoms, fever, and changes in taste and smell as the most common, but also extrapulmonary complications including the cardiovascular, gastrointestinal, dermatological, and neurological systems (Long et al., 2022). The alteration of these systems can last for months in some patients with long-COVID syndrome. This syndrome is described as symptoms that occur beyond 3 months from the onset of COVID-19, last for at least 2 months, and cannot be explained by an alternative diagnosis (World Health Organization [WHO], 2021). The estimated prevalence ratio of persistent symptoms after the infection is 0.54 in hospitalized and 0.34 in non-hospitalized patients, with fatigue, with a prevalence of 0.23, being the most common symptom reported, followed by memory problems with an estimated prevalence of 0.14 (Chen et al., 2022).

Regarding the etiology of long-COVID, studies have not yet reached a definite conclusion, but researchers have drawn hypotheses about the physiological pathways that may lead to the direct consequences of the viral infection in combination with inflammatory or autoimmune responses. Thus, some of the etiological factors for long-term symptoms associated with COVID-19 are viral persistence, either SARS-CoV-2 or RNAemia in tissues, persistent abnormalities in immune cells, changes in the inflammatory response, reactivation of latent pathogens, or autoimmune antibody development (Mantovani et al., 2022).

Regarding the neuropsychological long-term alterations described in the long-COVID syndrome (Frontera et al., 2021; Graham et al., 2021), memory is the predominant function altered, but also executive functions and visuospatial function (Ardila and Lahiri, 2020; Beaud et al., 2021; Jaywant et al., 2021; Llana et al., 2022). In this sense, when assessing memory, most studies were designed to detect declarative memory impairment, and other memory systems were not so profoundly explored (Llana et al., 2022). Declarative memory consists of memory for events and facts that are stored and can be explicitly retrieved (Squire et al., 2004). Several neuropsychological tests have been used to investigate the effects of the virus on declarative memory, such as the 16-item Grober and Buschke Free/Cued Recall Paradigm, the Corsi Block Tapping test, and the Rey Auditory Verbal Learning Test. These studies have found impairment in long-term verbal and visuospatial memory, as well as verbal learning (Llana et al., 2022). The neuroanatomical bases of declarative memory rely on the medial temporal lobe, including the hippocampus and other structures of the limbic system, which participates in memory and emotion (Catani et al., 2013).

SARS-CoV-2 causes olfactory dysfunction in many patients, being reported by long-COVID patients as a frequent symptom (Doty, 2022). Some possible causes of olfactory dysfunction are olfactory cleft obstruction, olfactory bulb atrophy, inflammation, downregulation of olfactory receptor proteins, and massive activation of macrophages and release of cytokines (Keshavarz et al., 2021; Xydakis et al., 2021; Frosolini et al., 2022). The virus can enter the olfactory bulbs and affect the brain through transcribriform or vascular routes (Brann et al., 2020). Studies have described how the virus can infect microglia and astrocytes, causing activation of these glial cells, and this effect may affect communication between neurons and neurogenesis (Vargas et al., 2020). In fact, neurogenesis is altered in the hippocampus of patients and rodents infected by the virus (Soung et al., 2022). Neuroimaging studies have detected that the hippocampus, parahippocampal cortex, and amygdala, which are brain areas connected to the olfactory bulb, show degeneration and volume reduction in subjects suffering from mild COVID-19 infection (Douaud et al., 2022). Olfactory bulb dysfunction may extend to connected and proximal regions of the limbic systems that support memory (Kay, 2022). Studies that analyze the associations between symptoms and memory performance have found that olfactory dysfunction in long-COVID patients is frequently related to lower scores in tests assessing declarative memory (Damiano et al., 2022; Delgado-Alonso et al., 2022).

A different type of memory, which is supported by various brain systems, is procedural memory (Squire and Dede, 2015). This memory is not related to the limbic system function. The brain regions involved in procedural memory are the frontal and parietal cortices, the basal ganglia, and the cerebellum (Camina and Güell, 2017). Procedural memory is a type of implicit memory that aids the performance of specific tasks without conscious awareness of previous experiences, such as the stored motor programs of routine or well-rehearsed actions (Cubelli and Della-Sala, 2020). This memory has been poorly explored in long-COVID patients. Only studies assessing subjective complaints have reported forgetfulness related to how to do routine tasks in 15% of cases (Davis et al., 2021; Callan et al., 2022), and no studies to date have assessed this memory using objective measures of performance. Magnetic resonance imaging 2 weeks after hospital discharge in COVID survivors (Hafiz et al., 2022) or long-COVID patients (Besteher et al., 2022) showed basal ganglia and limbic system alterations in comparison with controls. These brain abnormalities were associated with fatigue symptoms in the post-acute phase (Hafiz et al., 2022).

**FIGURE 1:**
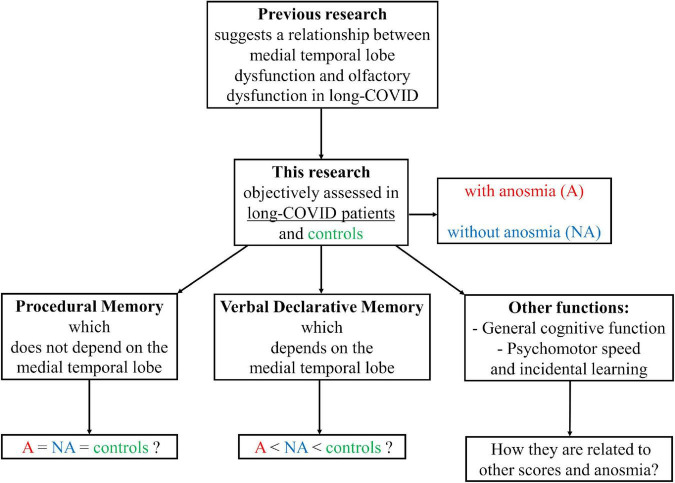
Research hypothesis diagram.

Previous research points to medial temporal lobe dysfunction in long-COVID patients and suggests a relationship between medial temporal lobe dysfunction and olfactory dysfunction in these patients. Also, no published studies assessed procedural memory in long-COVID patients with objective tests. This type of memory is anatomically dissociated from the medial temporal lobe. This study assessed verbal declarative memory, procedural memory, general cognitive function, psychomotor speed, and incidental learning in long-COVID patients with and without anosmia and healthy individuals. The principal aims of the study were: (i) to determine the characteristics of procedural memory and declarative memory in long-COVID patients compared to healthy people; (ii) to investigate whether anosmia has adverse effects on the cognitive skills studied; and (iii) to explore possible differences in the relationship between the performance on the tests assessing procedural and declarative memories and the performance on the tests measuring general cognitive function, psychomotor speed, and incidental learning, mediated by the presence or absence of anosmia or long-COVID syndrome (Figure 1).

## Materials and methods

### Participants

Forty-two long-COVID participants (4 male) were recruited from several Spanish-long-COVID associations (Aragón, Asturias, Galicia, and Valencia). These participants met the criteria for inclusion following the World Health Organization’s definition of long-COVID (World Health Organization [WHO], 2021): history of probable or confirmed SARS-CoV-2 infection with symptoms extending beyond 3 months from the onset of COVID-19, lasting for at least 2 months, and which cannot be explained by an alternative diagnosis. Participants were contacted via email and they were presented the study. Those who agreed to participate in the study completed online the Spanish adaptation of the National Health Service (NHS) Long COVID Pre- Assessment Questionnaire version 3 (National Health Service [NHS], 2021), that was used to explore clinical long-COVID symptomatology. Media was used for recruiting 30 additional healthy volunteers (12 male) that formed the control group (group C). Volunteers were invited through interviews on the radio, local newspapers and social media to contact via email with researchers of the study. Group C was included to obtain measures in a condition of health. All participants were Spanish native speakers without present or past severe neurological, psychological, or physical conditions or disorders that could potentially interfere with the results. Relevant sociodemographic information and clinical characteristics of the samples are shown and compared in Table 1.

**TABLE 1:** Demographic information and clinical characteristics of the sample related to the COVID history.

	Sample (*N* = 72)	Group A (*n* = 17)	Group NA (*n* = 25)	Group C (*n* = 30)	*P*
Sex (F, M; M%)	56, 16; 22.2%	15, 2; 11.8%	23, 2; 8.0%	18, 12; 40.0%	0.009^b^
Age (Years)^a^	43 (35–49)	42 (31–46)	47 (41–51)	40 (34–50)	0.097^c^
SES^a^	6 (6–8)	7 (5–8)	6 (5–7)	6 (6–7)	0.136^c^
Annual income^a^	30 (20–40)	30 (20–30)	30 (20–40)	30 (20–40)	0.767^c^
Handedness (*n*, %)					0.506^b^
Right-hand	67, 93.1%	17, 100%	22, 88%	28, 93.3%	
Left-hand	4, 5.6%		2, 8%	2, 6.7%	
Ambidextrous	1, 1.4%		1, 4%		
Ethnicity (*n*, %)					0.316^b^
White	69, 95.8%	17, 100%	24, 96%	28, 93.3%	
Bla., Lat., Car., Afr.	2, 2.8%			2, 6.7%	
Prefer not to say	1, 1.4%		1, 4%		
BMI^a^	24 (22–29)	28 (22–31)	23 (21–27)	23 (21–27)	0.371^c^
ED Diagn. (*n*, %)	9, 12.5%	0, 0%	7, 28.0%	2, 6.7%	0.012^b^
**Acute phase of COVID**					
Hospit. (*n*, %)	9, 21.4%	3, 17.6%	6, 24%	N/A	0.622^b^
Vent. assist. (*n*, %)					0.486^b^
No	35, 83.3%	15, 88.2%	20, 80%	N/A	
Intubated	2, 4.8%		2, 8%	N/A	
Enhanced RS	5, 11.9%	2, 11.8%	3, 12%	N/A	
**Long-COVID symptoms**					
Months^a^	16 (8–19)	18 (5–22)	15 (10–18)	N/A	0.389^d^
≤6 (*n*)	9	5	4		
7–12 (*n*)	4	0	4		
13–18 (*n*)	18	7	11		
≥19 (*n*)	11	5	6		
Sense of taste					<0.001^b^
Ageusia (*n*, %)	9, 21.4%	8, 47.1%	1, 4%	N/A	
Metal. taste (*n*, %)	6, 14.3%	4, 23.5%	2, 8%	N/A	
Sleep disturb. (*n*, %)	33, 78.6%	14, 82.4%	19, 76%	N/A	0.622^b^
Nightmares (*n*, %)	21, 50%	5, 29.4%	16, 64%	N/A	0.028^b^
Cog. disturb (*n*, %)	42, 100%	17, 100%	25, 100%	N/A	N/A
Brain fog (*n*, %)	39, 92.9%	16, 94.1%	23, 92%	N/A	0.794^b^
Fatigue (*n*, %)	39, 92.9%	17, 100%	22, 88%	N/A	0.138^b^
Headache (*n*, %)	27, 64.3%	13, 76.5%	14, 56%	N/A	0.174^b^
Vis. disturb. (*n*, %)	32, 76.2%	13, 76.5%	19, 76%	N/A	0.972^b^
Rec. fevers (*n*, %)	9, 21.4%	5, 29.4%	4, 16%	N/A	0.298^b^
Myalgia (*n*, %)	36, 85.7%	14, 82.4%	22, 88%	N/A	0.608^b^
Joint pain (*n*, %)	37, 88.1%	14, 82.4%	23, 92%	N/A	0.343^b^
Chest pain (*n*, %)	24, 57.1%	9, 52.9%	15, 60%	N/A	0.650^b^
Tinnitus (*n*, %)	23, 54.8%	10, 58.8%	13, 52%	N/A	0.663^b^

^a^Data are shown as median (first quartile–third quartile); ^b^Pearson chi-squared test; ^c^Kruskal–Wallis test; ^d^Mann-Whitney *U*-test. All the participants had >12 years of education.

SES, subjective socio-economic status (scale range from 1 to 10 points). Annual income is reported on a 5-point scale (ranging from 10 to 50 thousand euros). Bla., Lat., Car., Afr., Black, Latino, Caribbean or African; BMI, body mass index; ED diagn., emotional disorder diagnosis; Hospit., hospitalization; N/A, not applicable; Vent. assist., ventilatory assistance; RS, respiratory support; Months, months from diagnosis to assessment; Metal., metallic; Disturb., disturbance; Cog., cognitive; Vis., visual; Rec., recurrent.

This study was conducted in compliance with the European Community Council Directive 2001/20/EC and the Helsinki Declaration for biomedical research involving humans. The experimental data were collected after obtaining informed written consent from each subject. The study was approved by the local ethics committee.

### Olfactory function assessment

The experience of olfactory function was assessed using an online Spanish adaptation of the NHS Long COVID Pre- Assessment Questionnaire version 3 (National Health Service [NHS], 2021). This was applied to long-COVID participants. In this questionnaire participants reported their original/acute COVID symptoms and long-COVID symptoms. They also answered to the Yes/No question “Do you have anosmia (“no sense of smell”)?”

The sample of long-COVID patients was divided into two groups based on the presence or absence of anosmia in their reports. Patients of group A (*n* = 17) reported anosmia, while patients of group NA (*n* = 25) did not report anosmia.

The sample was also divided into 4 groups according to the months elapsed from the COVID diagnosis to assessment (groups: ≤ 6-months, 7–12-months, 13–18-months, and ≥ 19-months; Table 1).

### Procedural memory assessment

Procedural memory was assessed with the Mirror Tracing Test (MTT; Milner, 1962) (Model 58024E, Lafayette Instrument, USA), which measures the capacity to adapt to a novel trajectory within a sequence of practiced movements (Laforce and Doyon, 2002). MTT is an apparatus that contains a metal platform attached to a metal plate and a vertically hinged mirror. On the metal platform, there is the outline of a six-pointed star-shaped figure. The apparatus has a metal pen connected to the platform and to an automatic error-counter.

In each session, participants were seated in front of the MTT and prevented from seeing the six-pointed star-shaped figure directly by adjusting the metal plate. Thus, the star could only be seen through the vertical mirror. An investigator instructed the participants to trace the star with their dominant hand as quickly and accurately as possible while avoiding errors and remaining quiet. On each assessment day, participants were required to carry out four trials. Trials 1–4 (T1–T4) were completed on the first assessment day (Day 1), and trials T5–T8 on the following day (Day 2). Each trial had to be completed in a maximum of 10 min with a 10-s inter-trial interval in each session. The error rate (ER) was the total number of times the participant traced inside or outside the boundary lines of the star and was automatically recorded in each trial. Time per trial (TPT) was registered by the investigator with a standard stopwatch. Both parameters were assessed for all participants during the eight trials.

To measure procedural learning, the first two trials of Day 1 (T1–T2) were contrasted with the last two trials of Day 1 (T3–T4). To measure the consolidation of procedural learning, the first two trials of Day 2 (T5–T6) were contrasted with the last two trials of Day 1 (T3–T4). We also obtained subject-specific performance indices (expressed as percentages) of ER (ERI) and TPT (TPTI) for procedural learning (Day 1: ERI-d1 and TPTI-d1) and consolidation of learning (Day 2: ERI-d2 and TPTI-d2), using the following formulas:

ERI-d1 and TPTI-d1:


$$\frac{\left(\mathrm{T3}+\mathrm{T4}\right)\;-\;\left(\mathrm{T1}+\mathrm{T2}\right)}{\left(\mathrm{T1}+\mathrm{T2}\right)}$$

ERI-d2 and TPTI-d2:


$$\frac{\left(\mathrm{T5}+\mathrm{T6}\right)\;-\;\left(\mathrm{T3}+\mathrm{T4}\right)}{\left(\mathrm{T3}+\mathrm{T4}\right)}$$

The indices were negative when participants improved their performance over a given period. If the indices were near zero or positive, it was considered that there was no improvement.

### Declarative memory assessment

Declarative memory was measured with the Spanish version of the Paired-Associate Learning (PAL) test from the Wechsler Memory Scale (WMS-III) (Wechsler, 1997). The task consists of eight paired-associate words with no semantic relationship, which must be learned, recalled, and recognized.

On Day 1, the paired words were learned in four learning trials (T1–T4). In each trial, the researcher read a list of eight paired words. After this, the participant performed an immediate cued-recall trial where the researcher presented the first word of each pair and requested the immediate recall of its paired word. The order of presentation of the paired words varied through the four learning trials. A score was obtained for each of the 4 immediate cued-recall trials (maximum score per trial: 8). The sum of the four scores was computed to obtain the total number of correctly recalled pairs (PAR-I, maximum score: 32). The learning index was also obtained (PALI). PALI is the number of paired words correctly recalled in the last immediate cued-recall trial (Trial 4) contrasted with the number of paired words correctly recalled in first immediate cued-recall trial (Trial 1) (range score: −8 to +8, a higher value indicates higher learning across the four trials). Then, after 25–35 min, delayed cued-recall (PAR-D) was requested (PAR-d1). In PAR-d1 (maximum score: 8), the researcher requested the cued-recall of the paired words, giving the first word of each pair as a cue. Next, a delayed recognition trial was conducted (PARe-d1). In PARe-d1 (maximum score: 24), the participant was requested to recognize the previously presented pairs of words in a list of 24 pair-associated words, composed of 12 previously presented pairs (four duplicated) and 12 distractors. On Day 2, 24 h later, cued-recall (PAR-d2) (maximum score: 8) and recognition (PARe-d2) (maximum score: 24) were requested in the same way as described for the PAR-d1 and PARe-d1 trials.

### Assessment of other cognitive abilities

The Spanish Version 8.1 of the Montreal Cognitive Assessment scale (MoCA; Nasreddine et al., 2005) was used to obtain a score of the overall level of cognitive abilities (maximum score: 30; cognitive impairment: <26).

Psychomotor speed, sustained attention, and incidental learning were measured with the Digit Symbol Substitution Test (DSST). This is a subtest of the Wechsler Adult Intelligence Scale-III (WAIS-III; Wechsler, 1997). The DSST is a paper-and-pencil cognitive test that presents a coding matrix containing the digits 1–9 paired with a symbol. On the same page, a series of digits with a blank space for sketching the symbol is presented. Participants are requested to do the task as fast as possible. There is a time limit of 120 s to match the symbols with their corresponding numbers. When participants do not complete the first four lines of the task on time, more time is given to complete the full four lines to ensure enough experience with digit-symbol pairing. The DSST score consists of the number of correctly matched symbols in 120 s (DSS-M, maximum score: 133). Immediately after completing the task, the researcher gives the participant a new sheet of paper with the digits 1–9 in two lines. Participants are required to complete the blank spaces by drawing from memory the symbols paired with each number, with no time limit. This cued-recall task provides a measure of incidental learning (DSS-IL, maximum score: 18). Subsequently, participants were asked to draw all the symbols they could remember in a free-recall test without digits. In this task, free-recall of the incidental learning was registered (DSS-R, maximum score: 9).

### Procedure

All participants individually completed the online sociodemographic questionnaire, and the long-COVID patients also completed the Long-COVID Pre-Assessment Questionnaire. Then, participants were scheduled separately to carry out the neuropsychological assessment in two consecutive sessions separated by 24 h (Day 1 and Day 2). On Day 1, participants completed the neuropsychological assessment in the following sequence: MTT, MoCA, PAL, DSST, delayed PAL tests. On Day 2, the sequence was: PAL and MTT. The session lasted no more than 45 min on Day 1 and no more than 20 min on Day 2. There was a 10-min rest between the MTT and MoCA on Day 1. Both sessions were held between 09:00 and 13:00 or between 16:00 and 20:00. Due to technical problems, 1 participant of group A, 2 participants of group NA and 1 participant of group C could not be assessed with the MTT.

### Statistical analysis

Most of the variables had a non-normal distribution after applying the Shapiro-Wilk test, so we used the Kruskal–Wallis test to compare the groups’ scores of the neuropsychological tests, and post-hoc multiple comparisons with Bonferroni correction when significant group effects were found. Additional Kruskal–Wallis tests compared the scores of the neuropsychological tests among the groups of patients divided according to the months elapsed from the infection to assessment (≤6, 7–12, 13–18, and ≥19-months). To study the relationship between the MoCA and DSST scores and the scores of the procedural and declarative memory tests (i.e., MTT and PAL) mediated by Group, non-parametric partial correlations were calculated separately for each group, considering these variables. All the correlations were calculated controlling for the variables Sex and Age. When a significant correlation coefficient was found, we tested significant differences in the coefficients between pairs of groups using Fisher’s *Z*-test (Hidalgo et al., 2014). All the analyses were performed with the IBM SPSS Statistics, Version 26 (IBM Corp.). The level of statistical significance was set at *P* < 0.05. We used Kirk (1996) and Cohen’s (1988) guidelines for the interpretation of the effect size of the Kruskal–Wallis tests, (η^2^ = 0.06 = 0.14 medium; η^2^ ≥ 0.14 = large), and the strength of the correlations, (*r* = 0.3–0.5 medium; *r* > 0.5 = large), respectively.

## Results

### Group differences in procedural memory

**FIGURE 2:**
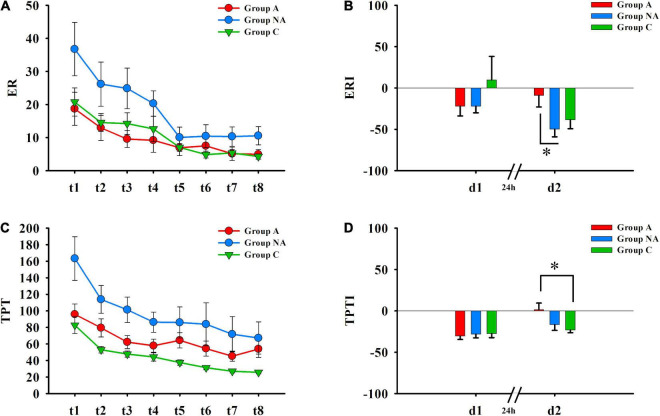
Procedural memory. **(A)** Error rates (ER) recorded during the eight trials (Day 1: t1–t4; Day 2: t5–t8) of the MTT. **(B)** Differences in ERI of day 1 (d1) and of day 2 (d2) among A, NA, and C groups (Kruskal–Wallis test, Bonferroni correction. *P = 0.043). **(C)** Time per trial (TPT) registered during the eight trials (Day 1: t1–t4; Day 2: t5–t8) of the MTT. **(D)** Differences in TPTI of day 1 (d1) and of day 2 (d2) among A, NA, and C groups (Kruskal–Wallis test, Bonferroni correction. *P = 0.022).

Table 2 shows the Kruskal–Wallis tests and the statistic *H*, with its degrees of freedom and significance. There were group differences in the performance indices of Day 2 (ERI-d2 and TPT-d2, *P*s ≤ 0.042, η^2^ = 0.07 and η^2^ = 0.08, respectively, Figure 2). As mentioned, the higher these indices are, the less improvement they reflect. The post-hoc multiple comparisons with Bonferroni correction showed that the ERI-d2 index was higher in the participants of group A than in those of group NA (*P* = 0.043) but was similar in the participants of group C compared both to group A and group NA (*P*s ≥ 0.148, Figure 2). The TPTI-d2 index was higher in the group A than in the group C (*P* = 0.022) but was similar in group NA compared both to group A and group C (*P*s ≥ 0.184, Figure 2).

**TABLE 2:** Mean ± standard deviation of the study variables and group comparisons.

	Group A (*N* = 17)	Group NA (*N* = 25)	Group C (*N* = 30)	Kruskal–Wallis test	
				*H*-value (df = 2)	*P*-value
MoCA	25.81 ± 2.42	26.39 ± 2.64	28.07 ± 1.58	14.46	**0.001**
**DSST**					
DSS-M	64.50 ± 16.08	69.96 ± 17.92	84.59 ± 10.64	16.91	**< 0.001**
DSS-IL	10.19 ± 4.79	10.35 ± 4.34	13.41 ± 3.71	7.70	**0.021**
DSS-R	6.81 ± 1.47	7.60 ± 0.99	7.83 ± 1.04	7.44	**0.024**
**MTT**					
ERI-d1	−21.98 ± 47.19	−21.85 ± 39.10	9.70 ± 153.19	0.01	0.994
ERI-d2	−9.01 ± 55.78	−49.81 ± 44.19	−38.37 ± 58.68	6.35	**0.042**
TPTI-d1	−30.41 ± 16.59	−28.08 ± 22.01	−27.37 ± 26.84	0.06	0.972
TPTI-d2	1.39 ± 32.01	−16.59 ± 33.37	−23.01 ± 18.37	7.29	**0.026**
**PAL**					
PALI	4.44 ± 2.16	4.00 ± 1.93	3.76 ± 1.57	0.51	0.776
PAR-I	14.94 ± 4.61	18.26 ± 8.03	21.34 ± 6.90	11.87	**0.003**
PAR-d1	5.06 ± 1.88	5.48 ± 2.29	6.72 ± 1.90	12.08	**0.002**
PAR-d2	4.94 ± 1.91	5.09 ± 2.29	6.52 ± 1.96	10.53	**0.005**
PARe-d1	23.50 ± 0.82	23.35 ± 1.72	23.86 ± 0.35	3.54	0.170
PARe-d2	23.69 ± 0.60	23.04 ± 2.25	23.79 ± 0.49	2.45	0.293

Significant difference is in bold. Df, degrees of freedom; Group A, anosmia; Group NA, absence of anosmia; Group C, control; MoCA, Montreal Cognitive Assessment scale; DSST, digit symbol substitution test; DSS-M, matching task of the DSST; DSS-IL, incidental learning of the DSST; DSS-R, free recall test of the DSST; MTT, Mirror Tracing Test; ERI-d1 and ERI-d2, error rate index on Day 1 and Day 2, respectively; TPTI-d1 and TPTI-d2, time per trial index on Day 1 and Day 2, respectively; PAL, Paired-Associate Learning; PALI, learning index; PAR-I, immediate cued-recall; PAR-d1 and PAR-d2, delayed cued-recall on Day 1 and Day 2, respectively; PARe-d1 and PARe-d2 = recognition on Day 1 and Day 2, respectively.

### Group differences in declarative memory

**FIGURE 3:**
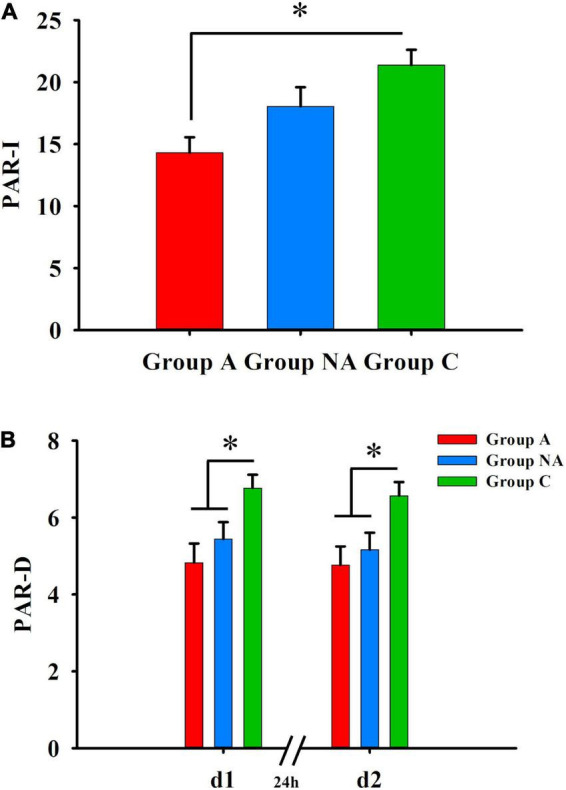
Declarative memory. **(A)** Differences in PAR-I among A, NA, and C groups (Kruskal–Wallis test, Bonferroni correction. *P = 0.002). **(B)** Differences in delayed cued-recall (PAR–D) PAR-d1 and PAR-d2 among A, NA, and C groups (Kruskal–Wallis test, Bonferroni correction. *Ps ≤ 0.045).

Table 2 shows the statistics of the group comparisons. The participants’ scores differed among the groups in the cued-recall tests (PAR-I, PAR-d1, and PAR-d2, all *P*s ≤ 0.005, η^2^ ≥ 0.13). The post-hoc tests showed that the participants of group A had lower scores than the participants of group C (*P* = 0.002), and the participants of group NA had similar scores as those of groups C and A (*P*s ≥ 0.222) in PAR-I (Figure 3). Besides this, the PAR-d1 and PAR-d2 scores were also lower in group A than in group C (*P* = 0.003 and *P* = 0.011, respectively; Figure 3). In addition, the PAR-d1 and PAR-d2 scores were lower in group NA than in group C (*P* = 0.045 and *P* = 0.037, respectively; Figure 3).

### Group differences in other cognitive abilities

**FIGURE 4:**
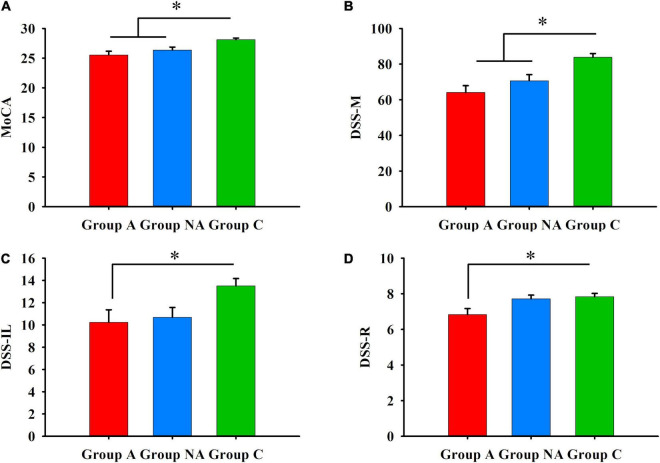
**(A)** General cognition. Differences in MoCA among A, NA, and C groups (Kruskal–Wallis test, Bonferroni correction. *Ps ≤ 0.017). **(B)** Psychomotor speed. Differences in DSS-M among A, NA, and C groups (Kruskal–Wallis test, Bonferroni correction. *Ps ≤ 0.015). **(C)** Incidental learning. Differences in DSS-IL among group A, group NA, and group C (Kruskal–Wallis test, Bonferroni correction. *P = 0.043). **(D)** Incidental learning. Differences in DSS-R among A, NA, and C groups (Kruskal–Wallis test, Bonferroni correction. *P = 0.026).

Table 2 presents group differences in the scores of the MoCA and the DSST tests. The participants of group A had lower scores than the participants of group C in the MoCA (*P* = 0.001, η^2^ = 0.21, Figure 4) and in the DSST tests (DSS-M, DSS-IL and DSS-R: *P* < 0.001, η^2^ = 0.27, *P* = 0.043, η^2^ = 0.08, and *P* = 0.026, η^2^ = 0.08, respectively, Figure 4). Also, the participants of group NA had lower scores than the participants of group C in the MoCA (*P* = 0.017, Figure 4) and the DSS-M (*P* = 0.015, Figure 4). However, the score of the A and NA groups was similar in all the tests (*P*s ≥ 0.083), and the scores of the NA and C groups did not differ in the DSS-IL and DSS-R tests (*P*s ≥ 0.086; Figure 4).

### Scores of neuropsychological tests and months elapsed from the infection to assessment

The scores of the MoCA and DSST were similar among the groups of patients divided according to the months elapsed from the COVID diagnosis to assessment (MoCA: *H*(3) = 3.27, *P* = 0.35; DSS-M: *H*(3) = 0.37, *P* = 0.95; DSS-IL: *H*(3) = 0.03, *P* = 0.99; and DSS-R: *H*(3) = 2.51, *P* = 0.47). The same result was obtained when the scores of MTT and PAL were compared (ERI-d1 and ERI-d2: *Hs*(3) ≤ 0.48, *Ps* = 0.92; TPTI-d1 and TPTI-d2: *Hs*(3) ≤ 3.15, *Ps* ≥ 0.37; PALI: *H*(3) = 7.19, *P* = 0.06; PAR-I: *H*(3) = 2.24, *P* = 0.52; PAR-d1 and PAR-d2: *Hs*(3) ≤ 6.03, *Ps* ≥ 0.11; and PARe-d1 and PARe-d2: *Hs*(3) ≤ 2.51, *Ps* ≥ 0.47).

### Relationship between procedural memory and other cognitive abilities

Table 3 shows the correlation coefficients and their significant tests computed for each group. The scores of the ERI-d1 were negatively associated with the scores of the DSS-IL in group A (*P* = 0.040), showing that the lower the participants’ incidental learning in the DSST, the more the errors they made in the procedural memory index (i.e., a higher score in ERI reflects less improvement over trials). Fisher’s *Z*-test showed no differences between group A and group NA (*Z* = −1.32, *P* = 0.187) or group C (*Z* = −1.84, *P* = 0.066) in the correlation coefficient.

**TABLE 3:** Non-parametric partial correlations controlling for sex and age.

		Group A (df = 12)				Group NA (df = 19)				Group C (df = 25)			
		MoCA	DSS-M	DSS-IL	DSS-R	MoCA	DSS-M	DSS-IL	DSS-R	MoCA	DSS-M	DSS-IL	DSS-R
ERI-d1	*r*	–0.111	–0.212	**−0.553**	–0.475	0.048	0.153	–0.151	0.035	0.001	–0.219	0.002	–0.194
	*P*	0.705	0.468	0.040	0.086	0.837	0.509	0.514	0.881	0.996	0.273	0.992	0.333
ERI-d2	*r*	–0.159	–0.062	0.160	0.173	–0.036	–0.239	–0.083	0.015	0.210	0.057	0.020	–0.139
	*P*	0.587	0.833	0.586	0.554	0.877	0.296	0.721	0.948	0.293	0.777	0.921	0.490
TPTI-d1	*r*	–0.385	–0.112	–0.491	–0.444	–0.033	–0.008	–0.136	–0.159	–0.121	0.062	–0.057	–0.248
	*P*	0.174	0.703	0.075	0.112	0.889	0.973	0.558	0.492	0.548	0.758	0.779	0.212
TPTI-d2	*r*	0.056	–0.375	0.079	–0.062	–0.113	–0.196	0.027	0.025	0.083	0.185	0.063	0.036
	*P*	0.849	0.187	0.788	0.833	0.627	0.394	0.907	0.914	0.679	0.357	0.755	0.858
PALI	*r*	**0.640** ^a,b^	**0.536** ^b^	0.360	0.291	–0.354	–0.135	0.131	0.127	–0.281	0.240	0.038	0.308
	*P*	0.014	0.048	0.206	0.313	0.115	0.558	0.571	0.583	0.156	0.227	0.850	0.118
PAR-I	*r*	0.211	–0.020	0.279	0.414	**0.434**	0.282	0.133	**0.508**	0.280	0.152	0.325	0.025
	*P*	0.470	0.946	0.335	0.141	0.049	0.215	0.567	0.019	0.157	0.449	0.098	0.901
PAR-d1	*r*	**0.551**	0.217	0.232	0.331	**0.435**	**0.443**	0.289	**0.528**	0.232	0.161	0.331	0.202
	*P*	0.041	0.456	0.424	0.247	0.049	0.044	0.205	0.014	0.243	0.422	0.091	0.312
PAR-d2	*r*	**0.592**	0.162	0.409	**0.615**	**0.589**	**0.599**	0.253	**0.441**	0.143	0.210	0.379	0.217
	*P*	0.026	0.580	0.147	0.019	0.005	0.004	0.268	0.045	0.477	0.293	0.052	0.276
PARe-d1	*r*	0.006	–0.187	0.005	0.080	**0.589**	**0.517** ^b^	**0.529**	0.208	0.210	0.146	0.280	0.214
	*P*	0.985	0.521	0.986	0.786	0.005	0.016	0.014	0.367	0.292	0.468	0.157	0.285
PARe-d2	*r*	0.015	–0.151	–0.156	–0.308	**0.474** ^c^	**0.444**	0.333	0.203	–0.152	0.078	0.094	0.040
	*P*	0.959	0.606	0.595	0.285	0.030	0.044	0.141	0.377	0.448	0.698	0.642	0.844

Significant difference is in bold. Correlation coefficient significantly different: ^a^Group A vs. Group C; ^b^Group A vs. Group NA; ^c^Group NA vs. Group C. df, degrees of freedom; Group A, anosmia; Group NA, absence of anosmia; Group C, control; MoCA, Montreal Cognitive Assessment scale; DSS-M, DSS-IL, and DSS-R, matching task, incidental learning test and free recall test of the DSST, respectively; ERI-d1 and ERI-d2, error rate index of the MTT on Day 1 and Day 2, respectively; TPTI-d1 and TPTI-d2, time per trial index of the MTT on Day 1 and Day 2, respectively; PALI, learning index of the PAL; PAR-I, immediate cued-recall of the PAL; PAR-d1 and PAR-d2, delayed cued-recall of the PAL on Day 1 and Day 2, respectively; PARe-d1 and PARe-d2, recognition test of the PAL on Day 1 and Day 2, respectively.

### Relationship between declarative memory and other cognitive abilities

Table 3 presents the *r* and *P*-values computed for each group. The PALI scores were positively associated with the MoCA and DSS-M scores (*P* = 0.014 and *P* = 0.048, respectively) in the participants of group A. Fisher’s *Z*-test comparing the correlation coefficient between PALI and MoCA showed that the coefficient was higher in group A compared both to group NA (*Z* = 3.13, *P* = 0.002), and group C (*Z* = 3.00, *P* = 0.003). The coefficient between PALI and DSS-M was higher in group A than in group NA (*Z* = 2.15, *P* = 0.032) but was similar in groups A and C (*Z* = 1.07, *P* = 0.284).

Concerning the cued-recall tests, the PAR-I scores were positively related to the MoCA and the DSS-R scores (*P* = 0.049 and *P* = 0.019, respectively) in the participants of group NA. Fisher’s *Z*-tests failed to find differences between the coefficients for any comparison tested (coefficient between PAR-I and MoCA: *Z* ≤ 0.73, *P* ≥ 0.465; coefficient between PAR-I and DSS-R: *Z* ≤ 1.86, *P* ≥ 0.063). Besides, the PAR-d1 score was positively associated with the MoCA score in groups A and NA (all *P*s ≤ 0.049) and with the score of both the DSS-M and DSS-R in group NA (all *P*s ≤ 0.044). The correlation coefficient between PAR-d1 and MoCA was similar in all the groups (all Zs ≤ 1.16, *P*s ≥ 0.246). Also, the coefficient between PAR-d1 and DSS-M was similar in all the groups (all Zs ≤ 1.09, *P*s ≥ 0.275), and the same applied to the coefficient between PAR-d1 and DSS-R (all Zs ≤ 1.33, *P*s ≥ 0.183). In addition, the PAR-d2 scores were positively related to the scores of the MoCA in groups A and NA (all *P*s ≤ 0.026) and to the DSS-M and DSS-R scores (all *P*s ≤ 0.045) in group NA. The groups showed similar correlation coefficients regarding the *r*-value between PAR-d2 and MoCA (all Zs ≤ 1.85, *P*s ≥ 0.064) and between PAR-d2 and DSS-M (all Zs ≤ 1.46, *P*s ≥ 0.144) and DSS-R (all Zs ≤ 0.88, *P*s ≥ 0.379).

Regarding the tests of recognition, the PARe-d1 and PARe-d2 scores of the participants of group NA were positively associated with the MoCA (all *P*s ≤ 0.030) and DSS-M scores (all *P*s ≤ 0.044). The PARe-d1 score was also positively related to the DSS-IL score. Fisher’s *Z*-test yielded no differences between group NA and groups A (*Z* = 1.96, *P* = 0.050) or C (*Z* = 1.61, *P* = 0.107) in the correlation coefficient between PARe-d1 and MoCA. The coefficient between PARe-d2 and MoCA was higher in group NA than in group C (*Z* = 2.33, *P* = 0.019) and was similar in groups NA and A (*Z* = 1.46, *P* = 0.144). The coefficient between PARe-d1 and DSS-M was higher in group NA than in group A (*Z* = 2.23, *P* = 0.025) and was equal in groups NA and C (*Z* = 1.48, *P* = 0.138). All the groups had similar *r*-values of the association between PARe-d2 and DSS-M (all Zs ≤ 1.84, *P*s ≥ 0.066) and between PARe-d1 and DSS-IL (all Zs ≤ 1.71, *P*s ≥ 0.087).

## Discussion

This study objectively assessed long-COVID performance in dissociated memory systems, including olfactory dysfunction as a relevant symptom. It is the first work to evaluate consolidation of declarative and procedural learning in long-COVID. Results revealed that long-COVID participants, regardless of the presence or absence of anosmia, had lower cognitive ability than controls when assessed with the MoCA. Lower psychomotor speed and sustained attention than controls were also observed in all long-COVID participants when evaluated with DSST. However, the incidental learning score in DSST, both cue-guided and free-recalled, was exclusively altered in participants with anosmia compared to controls. In addition, both the MoCA and DSST scores were related to declarative memory function exclusively in the long-COVID groups, but not in healthy participants, who did not show altered memory processes and presented greater score homogeneity. When both acquisition and consolidation of explicit/declarative and implicit/procedural memory were assessed in long-COVID patients, we found that the long-term retention of both memories was more vulnerable than their acquisition. Acquisition was only negatively affected in participants with anosmia when compared to healthy subjects in the immediate recall of declarative memories. Also, long-COVID participants presented more impairment in cued-recall of declarative memory than in tests of recognition memory, which was preserved. The alteration of cued-recall declarative memory was independent of whether the test delay was short (i.e., 25–35 min) or long (i.e., 24 h). In addition, anosmia was linked to lower procedural memory when assessed during long delay. This symptom is also very relevant when we evaluated the immediate cued-recall of declarative memory, as only the participants with anosmia showed worse performance than the controls. However, all the patients with long-COVID syndrome, regardless of the presence or absence of anosmia, had worse performance than controls in the delayed versions of the cued-recall tests.

When assessing anosmia symptoms in the long-COVID sample, 40% of participants reported anosmia. Fernández-de-Las-Peñas et al. (2021) reviewed the prevalence of symptoms at onset and post-COVID when they were reported by both hospitalized and non-hospitalized adult patients. Data synthesis of the reviewed studies revealed that the pooled prevalence of anosmia is 45.7% (Fernández-de-Las-Peñas et al., 2021). The same pooled prevalence was reported in the review of Narayanan et al. (2022), showing that Europe, America, and Middle East present higher prevalence of olfactory dysfunction than Asia and Africa. Some of the studies performed in Europe that have used questionnaires and interviews including questions about the presence or absence of olfactory dysfunction in COVID-19 reported an anosmia prevalence of 33.9% (Giacomelli et al., 2020), 47% (Klopfenstein et al., 2020), 49% (Singer-Cornelius et al., 2021) 64% (Spinato et al., 2020), 65% (Menni et al., 2020), and 86% (Lechien et al., 2020b). When studies used olfactory psychophysical tests, they reported anosmia prevalence of 39% (Singer-Cornelius et al., 2021), 48% (Lechien et al., 2020a), and 67% (Vaira et al., 2020). Studies indicate that there is discrepancy between the results obtained with objective tests and subjective reports (Lechien et al., 2020a; Singer-Cornelius et al., 2021). There is a tendency to overestimate olfactory dysfunction when it is subjectively reported (Lechien et al., 2020a; Singer-Cornelius et al., 2021). The prevalence of subjectively reported anosmia in the present study was lower than that found in most of the studies described above, even considering studies that evaluate this symptom objectively.

Our results show that long-COVID participants, all adults aged under 51 years, presented lower scores than controls in general cognition. However, although situated at the suggested cut-off of 26 points (Nasreddine et al., 2005), their scores could not be considered indicative of abnormal cognitive performance. The memory impairment we observed when we evaluated declarative memory retrieval could be reflected in this global index. In fact, the general index of cognition obtained from the MoCA includes an assessment of short-term memory and working memory, and both are types of declarative memories. Therefore, associations between MoCA scores and PAL performance in long-COVID participants are not surprising.

Long-COVID participants presented worse psychomotor speed and sustained attention than controls, as shown when they performed the first part of the DSST test. These functions were assessed by DSST, which is sensitive to the presence of cognitive dysfunction in a wide range of clinical populations (Jaeger, 2018). However, this test may lack specificity in terms of the cognitive functions tested. Performance on DSST requires several cognitive functions, including planning, working memory, motor speed, attention, and visuoperceptual functions (Jaeger, 2018). In addition, associative learning, required for paired learning, could also affect performance on the first part of the DSST test by increasing speed. Associative learning may also contribute to incidental learning when the individual remembers the pairs of symbols and numbers required in the second part of the task by both cued-guided recall and free recall (Jaeger, 2018). Therefore, it is not surprising that we found a relationship between performance on the PAL test, which requires learning and recall of pairs of words, and performance on DSST in long-COVID groups. The DSST has not been used previously to assess incidental learning after COVID-19 infection. The only study that has used this test evaluated exclusively digit-symbol pairing in recovered patients at a 1-month follow-up, finding impairment in this test (Gouraud et al., 2021). It is important to mention that the participants of this study differed from those of our study, as they did not meet the criteria for long-COVID diagnosis. However, psychomotor speed was altered in long-COVID patients with relevant neuropsychological symptoms or severe acute infection when assessing processing-speed deficits and sustained attention with the Symbol Digit Modalities Test (SDMT; Smith, 2007; Ferrucci et al., 2021; Ferrando et al., 2022) or other tests that provide an index of these functions (García-Sánchez et al., 2022; Vannorsdall et al., 2022; Zhao et al., 2022). From our DSST results, we can conclude that incidental learning, both cue-recalled (i.e., DSS-IL) or free-recalled (DSS-R), was exclusively altered in long-COVID participants who presented anosmia. Thus, the presence of anosmia, which is also a relevant factor accounting for difficulties in the immediate cued-recall of the PAL test, could reflect a greater vulnerability of brain regions involved in declarative learning. Declarative learning involves brain regions of the limbic system located in the medial temporal lobe (Clark et al., 2018). These structures, in turn, are closely related to olfactory dysfunction in COVID-hyposmia patients (Douaud et al., 2022; Morbelli et al., 2022).

This is the first study assessing implicit procedural learning in long-COVID. Previous literature has only mentioned the low prevalence of patients’ self-reported difficulty to perform routine tasks (Davis et al., 2021; Callan et al., 2022). Results of procedural memory assessed with the MTT show that only the consolidation of procedural learning was affected in long-COVID participants presenting anosmia, with no differences between long-COVID and healthy subjects in the acquisition of this implicit memory. This highlights the importance of this symptom, which contributes exclusively to long-term procedural memory deficits, allowing us to propose the hypothesis about the specific association of olfactory dysfunction with the impairment of brain regions of the medial temporal lobe, such as the hippocampus. In fact, the role of the hippocampus in the consolidation of procedural memories, that may initially require the involvement of the cerebellum or basal ganglia (Krakauer and Shadmehr, 2006), was revealed in a motor sequence task, similar to the MTT, which was performed one day after learning acquisition (Tucker et al., 2011; Schapiro et al., 2019). This means that basal ganglia and cerebellar cortex are involved in the initial storage of specific procedural memory tasks. However, this memory might be supported by other brain regions of the limbic system over time. Anosmia as a symptom in the long-COVID syndrome is frequently associated with the limbic system, both functionally (Damiano et al., 2022; Delgado-Alonso et al., 2022; Kay, 2022; Voruz et al., 2022; Yus et al., 2022) and structurally (Douaud et al., 2022; Morbelli et al., 2022). Therefore, a specific alteration of consolidation of procedural memory in long-COVID patients suffering from olfactory dysfunction is plausible. Studies assessing olfactory function in long-COVID have found that reported mental clouding was associated with more severe olfactory loss (Di Stadio et al., 2022). In addition, as in our study, olfactory loss was associated with cognitive impairment objectively assessed with neuropsychological tests of declarative memory (Damiano et al., 2022; Delgado-Alonso et al., 2022; Fiorentino et al., 2022).

When assessing verbal declarative memory, long-COVID participants presented more impairment in both delayed and long-term cued-recall tests than in recognition tests. This is consistent with previous research in long-COVID patients reporting impairment in verbal learning and verbal long-term memory when they were assessed with recall tests but not recognition tests of previously learned verbal material (Albu et al., 2021; García-Sánchez et al., 2022). However, other authors found that not only learning and recall but also verbal recognition, assessed with computerized tests, were impaired both in severe and mild long-COVID patients who presented temporal brain volume reduction (Widmann et al., 2021). Anosmia is relevant not only in the long-term recall but also in the immediate cued-recall of paired verbal items, suggesting that participants with this symptom are also vulnerable to an immediate evocation of verbal associations. The relationship between the difficulties in immediate verbal evocation and anosmia again shows that the presence of this symptom may indicate a higher predisposition to medial temporal lobe dysfunction, as the hippocampus is crucial both in the recognition and recall of declarative memories (Stark and Squire, 2000).

The findings of this study have implications for clinical practice. Long-COVID patients presented lower scores than controls in MoCA, but these scores were situated at the suggested cut-off of 26 points and considered indicative of normal cognitive performance. Therefore, a screening test, such as MoCA might fail to detect neuropsychological deficits in this population. In the light of the previously discussed results, the assessment protocol to detect cognitive deficits in this population should include declarative tests of long-term recall.

Strengths of this study are summarized in the following lines. The present study objectively assessed both procedural and declarative memory systems, as well as incidental learning, using neuropsychological tests in long-COVID patients who were assessed 3–28 months after COVID infection. This research is the first to compare procedural and declarative memories of long-COVID patients grouped on the basis of the presence or absence of olfactory dysfunction. The study not only examined learning, recall, and recognition memory processes, but it also evaluated long-term memory 24 h after acquisition. Finally, the study included a control group, consisting of participants without long-COVID, making possible to infer about the relative contribution of the infection to neurocognitive symptoms over and above the psychosocial effects related to the pandemic.

This study has some limitations. First, we do not know the pre-COVID neuropsychological function of the participants enrolled in this study. Thus, we cannot draw conclusions about a causal relationship between olfactory dysfunction and declarative and/or procedural memory impairment. Second, long-COVID participants in this study were evaluated 3–28 months after the acute phase of the COVID-19 infection by a subjective report of symptoms. Therefore, the characterization of anosmia was not provided by a standardized objective protocol. This report might be influenced not only by memory function but also by the individual’s subjective perception. This limitation also applies to the reported symptoms at the time of assessment, which were not objectively assessed. Third, given the voluntary participation in the study, some subjects with a higher degree of long-COVID symptoms may have been less prone to accept enrollment in the study. Therefore, our findings cannot be generalized to the entire COVID-19 population.

In conclusion, the results of this research support that the consolidation of both procedural and declarative memories is more affected than the acquisition of these memories in long-COVID, which is also a clinical condition more vulnerable to deficits in delayed recall than in recognition of declarative memories. Assessment of explicit and implicit memories 24 h after acquisition reveals difficulties in memory consolidation in the long-COVID group compared to controls. This alteration in the consolidation of procedural memory is especially relevant in those long-COVID participants with associated anosmia, who also are more vulnerable to deficits in immediate recall of verbal declarative memory. This suggests that anosmia in COVID-19 could be associated with long-term limbic system dysfunction.

## Data availability statement

The raw data supporting the conclusions of this article will be made available by the authors, without undue reservation.

## Ethics statement

The studies involving human participants were reviewed and approved by Comité de Ética de la Investigación de la UPV (P04_16_02_2022). The patients/participants provided their written informed consent to participate in this study.

## Author contributions

M-CJ, MM, and MM-L conceived and planned the experiments. M-CJ acquired the funding and administrated the project. CZ, MM, MM-L, and TL carried out the experiments. MM-L and TL contributed to the creation of the database. MM-L analyzed the data. CF and CZ designed the graphic representation. All authors drafted and reviewed the manuscript and approved its final version to be published and agreed to be accountable for all aspects of the manuscript.
